# Impact of Greenspace and Air Pollutants on Women During Menopause

**DOI:** 10.1093/geroni/igaf122.1803

**Published:** 2025-12-31

**Authors:** Sneha Dhanavanthri Muralidhara, Candace Brown

**Affiliations:** The University of North Carolina at Charlotte, Charlotte, North Carolina, United States; University of North Carolina, University of North Carolina, Charlotte, North Carolina, United States

## Abstract

Previous research has highlighted the diverse experiences of women during menopause, revealing significant variations in health outcomes both during midlife and later years. While much attention has been given to the role of individual lifestyle and social factors in contributing to these disparities, the impact of environmental factors on menopause and related health outcomes among middle-aged women remains limited. This narrative review aimed to summarize and interpret the literature on the environmental impact of greenspace, air pollution, and particulate matter on menopause and related health outcomes among middle-aged women. A systematic search of PsychInfo, Pubmed, Web of Science, CINAHL, and ProQuest was conducted for relevant scientific literature published between January 2014 and November 2024. Following inclusion and exclusion criteria, 18 articles were selected out of 9805 articles obtained. Emergent themes related to health and women’s aging included: 1) impact of greenspace; 2) air pollution and cardiovascular health; and 3) air pollution and the evolution of menopause. Greenspace was more beneficial for women with lower socioeconomic status (SES) and worse health status than those with higher SES. Higher exposure to resident ambient particulate matter was associated with accelerated atherosclerosis in women during early midlife, whereas long-term exposure contributed to elevated risk of atherosclerosis among post-menopausal women. Particulate matter exposure may also elevate the risk of cardiovascular disease in menopausal women, thereby accelerating aging. Inconsistent findings, due to diverse methods employed, require inquiry to further understand and identify environmental factors that substantially contribute to menopause and related outcomes.

